# Transplantation of a bone marrow mesenchymal stem cell line increases neuronal progenitor cell migration in a cerebral ischemia animal model

**DOI:** 10.1038/s41598-018-33030-9

**Published:** 2018-10-08

**Authors:** Yuri Shiota, Atsushi Nagai, Abdullah Md. Sheikh, Shingo Mitaki, Seiji Mishima, Shozo Yano, Md. Ahsanul Haque, Shotai Kobayashi, Shuhei Yamaguchi

**Affiliations:** 1 Clinical Laboratory Division, Shimane University Hospital, 89-1 Enya Cho, Izumo, 693-8501 Japan; 2 Department of Laboratory Medicine, Shimane University School of Medicine, 89-1 Enya Cho, Izumo, 693-8501 Japan; 3 Department of Internal Medicine III, Shimane University School of Medicine, 89-1 Enya Cho, Izumo, 693-8501 Japan; 4 Shimane University, 89-1 Enya Cho, Izumo, 693-8501 Japan

## Abstract

Mesenchymal stem cell (MSC) transplantation is demonstrated to improve functional and pathological recovery in cerebral ischemia. To understand the underlying mechanism, we transplanted a MSC line (B10) in a rat middle cerebral artery occlusion (MCAO) model and checked the proliferation and migration of neuronal progenitor cells (NPCs). B10 transplantation increased NPCs in the subventricular zone and their migration towards the lesion area at an earlier time. Fourteen days after MCAO, some NPCs were differentiated to neurons and astrocytes. Although B10 transplantation increased total number of both astrocytes and neurons, it only increased the differentiation of NPC to astrocyte. The mRNA of polysialylation enzyme ST8SiaIV and a chemokine SDF-1 were persistently increased in B10-transplanted groups. SDF-1-positive cell number was increased in the core and penumbra area, which was expressed in macrophage/microglia and transplanted B10 cells at 3 days after MCAO. Furthermore, SDF-1 mRNA expression in cell culture was high in B10 compared to a microglia (HMO) or a neuronal (A1) cell line. B10 culture supernatant increased *in vitro* A1 cell migration, which was significantly inhibited by siRNA-mediated SDF-1 silencing in B10. Thus, our results suggested that MSC transplantation increased endogenous NPC migration in cerebral ischemic condition by increasing chemokine and polysialylation enzyme expression, which could be helpful for the restorative management of cerebral ischemia.

## Introduction

In cerebral ischemic condition, sudden and severely compromised blood supply in a focal area causes necrotic death of brain tissue. Consequently, a neuroinflammatory process is initiated, leading to accumulation and activation of immune cells, and increased expression of several cytokines, chemokines, proteases and reactive oxygen species^1^. Such activation of immune system results further cell death in the peri-infarct area that progresses at a slower pace^2,3^. On the other hand, reparative processes including clearance of cell debris, expression of neurotropic factors and formation of glial scar to wall-off the infarct area from viable tissue are also observed^4–6^. The balance of such inflammatory and reparative events ultimately determines the formation of a mature lesion.

In addition to inflammatory and reparative processes, a regenerative process might also be attributed^7^. For example, the proliferation of neural progenitor cells (NPCs) is increased in the sub-ventricular zone (SVZ) of human stroke patients as well as focal cerebral ischemia animal models; as evidenced by the presence of polysialylated neural cell adhesion molecule (PSA-NCAM) positive cells in the area^8,9^. PSA-NCAM positive cells are considered as migrating NPCs^10,11^. These newly proliferated NPCs are suggested to migrate toward the lesion areas^12^, and differentiate into mature neurons^13^. However, such endogenous regenerative capacity of the brain seems to be insufficient to resolve the brain damage. Nevertheless, the strategy to boost up the regenerative capacity by increasing the proliferation and migration of endogenous NPCs could be promising targets for the therapy of cerebral ischemic condition.

Although much is known about the pathophysiology of cerebral ischemic condition, only available disease modifying treatment is the re-establishment of circulation with tissue plasminogen activator (tPA) or mechanical restoration of blood supply^14^. However, only a small proportion of the patients could receive tPA reperfusion therapy due to short treatment window and other factors^15^, signifying a necessity to improve the management system based on disease pathophysiology. Currently, a growing number of reports are suggesting that  the modulation of immune system, and regeneration and replacement of damaged brain tissue could be the potential targets for the disease management system^16–18^. For regenerative therapy, the strategy to increase the proliferation and migration of endogenous NPCs, and exogenous transplantation of stem cells including neural stem cells (NSCs), embryonic stem cells, induced pluripotent cells (iPS) and mesenchymal stem cells (MSCs) are under intense investigation^19–22^. Among the cells used for exogenous transplantation in cerebral ischemic condition, MSCs attract interest for its easy availability from various sources, and immunomodulatory and neuronal differentiation properties^18,23,24^. In previous studies, we have demonstrated that after transplantation in an animal stroke model, a mesenchymal stem cell line (B10) migrates selectively to the ischemic lesion areas and promote functional improvement^19^. As possible mechanisms of such beneficial effect, we have found that B10 transplantation modulates neuroinflammatory system and increases the expression growth factors including epidermal growth factor (EGF), basic fibroblast growth factor (bFGF) and insulin-like growth factor-1 (IGF-1)^19^. Since these growth factors plays an important role in NPC proliferation^25–27^, we hypothesized that B10 transplantation might increase neurogenesis in middle cerebral artery occlusion (MCAO) model. In this study, we have investigated NPC proliferation along with its migration towards the lesioned area of MCAO model animals. Moreover, we tried to elucidate the underlying mechanism of increased migration of NPC. Our results suggest that B10 transplantation increases NPCs proliferation and migration by regulating the expression of several proliferation and migration regulatory genes.

## Experimental Procedure

### Cell culture

The permission to use embryonic tissues and the procedures were approved by the Clinical screening committee for research involving human subjects and the Ethics Committee of Faculty of Medicine, the University of British Columbia, Canada. All experimental procedures involving human tissue were done with informed consents, and according to the guidelines approved by the Ethics Committee of Faculty of Medicine, the University of British Columbia. A human mesenchymal stem cell line (B10) was generated from human fetal bone marrow cells by stable transfection of viral oncogene v-*myc*^23^. B10 cells were cultured in complete MF^®^ medium (Toyobo, Osaka, Japan) containing 1% FCS and growth factor supplement. The cell line shows similar morphological and expressional phenotype, and differentiation potentials as primary human MSCs^23^.

A neuronal hybridoma cell line (A1) was generated by somatic fusion of a human fetal neuron and a human neuroblastoma cell^28^. A1 cells show similar morphological, electrophysiological and expressional phenotype as neuron. A1 cells were cultured in 5% FBS (Gibco, Invitrogen, Carlsbad, CA, USA) containing DMEM (Gibco).

A human microglia cell line (HMO6) was generated from human fetal primary microglia by stably transfected viral oncogene v-*myc*^29^. HMO6 cells were cultured in 5% FBS (Gibco) containing DMEM (Gibco).

### Transient focal cerebral ischemia animal model

All experimental procedures and protocols were approved by the Ethical Committee of Shimane University Faculty of Medicine, Shimane, Japan. All animal experimental procedures were in accordance with the guidelines and the regulations of the Institute of Experimental Animals, Shimane University, Shimane, Japan. Adult male Sprague-Dawley rats at the age of 7–8 weeks, weighing about 220 gm to 280 gm were used to generate transient focal cerebral ischemia model. The model was generated by transiently occluding middle cerebral artery, following previously described method^19^. Briefly, the rats were anesthetized with 4% halothane, and the right common, external and internal carotid arteries were exposed. Then a 4–0 monofilament nylon suture coated with silicon was introduced through external carotid artery, and passed through internal carotid artery until it blocked the origin of middle cerebral artery (MCA). Body temperature of the rats were maintained at 37± 0.3 °C during surgical procedure by means of a feedback heating system. After 90 min occlusion of MCA, the rats were re-anesthetized, and the nylon thread was removed to restore blood flow. The average mortality rate of MCAO rats within the experimental period was about 25%.

### B10 transplantation

After 24 h of transient MCAO, all animals were evaluated neurologically using a modified neurological scoring system (mNSS). The total score for the test was 22 points, where increasing score indicates the severity of injury^19^. The details of mNSS scoring system are described in Supplemental Table 1. Previously, we have found that rats with 14–18 points of mNSS had a stable stroke volume^19^. Rats with 14–18 points were assigned randomly to the following two groups, (1) PBS control (received phosphate-buffered saline (PBS)), and (2) B10 transplanted group (received B10). To transplant B10, the rats were anesthetized with 4% halothane, and 3 × 10^6^ cells in 100 μl PBS were injected through right jugular vein. PBS control group was injected with same volume of PBS following similar procedure.

### Immunofluorescence staining

Three, seven and fourteen days after MCAO (n = 5 in each group), the experimental rats were deeply anesthetized and transcardially perfused with 0.9% NaCl (normal saline), followed by 4% paraformaldehyde (PFA) solution in PBS (pH 7.4). Brains were removed, post-fixed in 4% PFA for 6 h, and then transferred to 20% sucrose overnight for cryoprotection. Then tissue blocks of 2 mm thickness were prepared by coronal section, and stored at −20 °C. For immunostaining, coronal sections of 10 μm thickness were cut with a cryostat. For blocking nonspecific binding, the tissue sections were treated with blocking buffer containing 5% normal horse or goat sera and 0.1% TritonX 100 for 30 min. Sections were incubated overnight at 4 °C with primary antibodies. Following primary antibodies were used: mouse anti-PSA-NCAM IgG (1: 100, Santa Cruz Biotechnology, Santa Cruz, CA), goat anti-SDF-1 IgG (1: 100, Santa Cruz), rabbit anti-β-tubulin IgG (1: 1000, Abcam), GFAP (DAKO, Glostrup, Denmark), mouse anti-rat CD68 IgG (1: 100, ED-1, Oxford Biotechnology, Oxford, UK) and mouse anti-human nuclei IgG (1: 100, Abcam). After washes with PBS, sections were incubated with appropriate species specific fluorescence-conjugated IgG (goat-anti-mouse IgG Texas red, goat anti-mouse IgG Texas FITC, goat anti-rabbit Texas red, goat anti-rabbit IgG FITC, or donkey anti-goat IgG FITC, 1:200, Santa Cruz) for 1 h. Hoechst 33258 (Sigma, St. Louis, MO) staining was done to identify nuclei of the cells. After staining, sections were mounted with Ultramount (DAKO) and photographed with a fluorescence microscopy system (Nikon E600). For immunohistochemical analysis, immunostained sections were examined blindly by 2 investigators (YI and AN). Cell counting was done in 3 consecutive sections of 2 mm apart in 5 random fields of designated areas at X400 magnification.

### *In vitro* migration assay

A1 cell migration in response to B10 culture supernatant was assessed using a 48-well microchemotaxis chamber (Neuroprobe, Cabin John, MD) and a 5-μm pore membrane (Costar, High Wycombe, England), as described previously^30^. The bottom surface of the membrane was coated with fibronectin (6.5 μg/ml, Sigma). B10 or A1 cells were cultured in 0.5% FBS containing DMEM for 24 h, and the culture supernatant was collected. Twenty-eight μl of culture supernatant was added in triplicate into the lower wells of the chemotaxis chamber. The filled lower chamber was then overlaid with the coated membrane, and the top chamber was assembled to form wells. Fifty microliters of A1 cell suspension (5 × 10^5^/ml) in DMEM containing 0.5% FCS was added to each of the wells. After incubation for 2 h, the migrated cells on the membrane were fixed with methanol and stained with Harris’ hematoxylin (Sigma). Migration was assessed by counting migrated cells in five microscopic fields per well at 400 × magnification. DMEM-only and DMEM containing 0.5% FBS were used as control.

### SDF-1 gene silencing

Silencing SDF-1 mRNA expression was done by transfecting gene-specific silencing siRNA (Santa Cruz)^31^. SDF-1 siRNA was transfected to B10 cells using HiPerfect transfection reagent (Qiagen) according to the manufacturer’s protocol after optimizing the condition. A non-silencing siRNA (Qiagen) was also transfected to B10 cells, which served as negative control. Forty-eight hours after transfection, the silencing effects were checked by real-time PCR and SDF-1 immunocytochemistry. Then transfected cells were treated with 0.5% FBS containing DMEM for 24 h. The culture supernatant was collected and used for migration assay.

### Immunocytochemistry

B10 cells were seeded on an 8-well glass chamber slide. After overnight culture, the cells were transfected with SDF-1 siRNA, or non-silencing siRNA as described previously. Forty-eight hours after transfection, the cells were fixed with 100% methanol for 20 min. After wash, the cells were treated with a blocking solution containing 5% normal horse serum and 0.1% TritonX100 for 30 min. Then the cells were incubated with goat anti-SDF-1 IgG (1: 200; Santa Cruz) for 1 h at room temperature, followed by donkey anti-goat IgG FITC (1:200, Santa Cruz) for 1 h at room temperature. Hoechst 33258 (Sigma) staining was done to identify nuclei of the cells. After staining, sections were mounted with Ultramount (DAKO) and photographed with a fluorescence microscopy system (Nikon E600). The fluorescence signals of immunoreactive protein was quantified by ImageJ software.

### Real time RT-PCR

Three, seven and fourteen days after MCAO (n = 5 in each group), the rats were deeply anesthetized and sacrificed. The brains were immediately removed and sectioned on ice, and the areas including infarct core, penumbra and contra lateral cortex were identified macroscopically. Then the brain tissues of above mentioned areas were collected in RNAlatter reagent (Applied Biosystems, Warrington, UK), and stored at −80°C. Total RNA was isolated from the brain tissues using Trizol reagent (Invitrogen, Carlsbad CA) according to the manufacturer’s protocol. To prepare first strand cDNA, 2 μg of total RNA was reverse transcribed with reverse transcriptase enzyme (RiverTraAce, Toyobo, Osaka, Japan) in a 20 μl of reaction mixture following the manufacturer’s instructions.

For quantification of mRNA, a SyBr green-based real time PCR system (Applied Biosystems, Warrington, UK) was employed, where gene specific primers were used to amplify specific cDNA. Quantification of mRNA was calculated using a cDNA sample as a calibrator. The quantified value of each sample was normalized with that of GAPDH value of same sample, which was amplified simultaneously with target gene. The list of primer sequences used for real time PCR is shown in Supplemental Table 2.

### Statistical analysis

Experimental data were analyzed by one-way ANOVA and means were tested with Student-Newman-Keuls test for the difference between the means. Statistically significant difference between mean values was set at *p* < 0.05.

## Result

### Effects of B10 transplantation on PSA-NCAM-positive cells accumulation in MCAO rat brain

**Figure 1:**
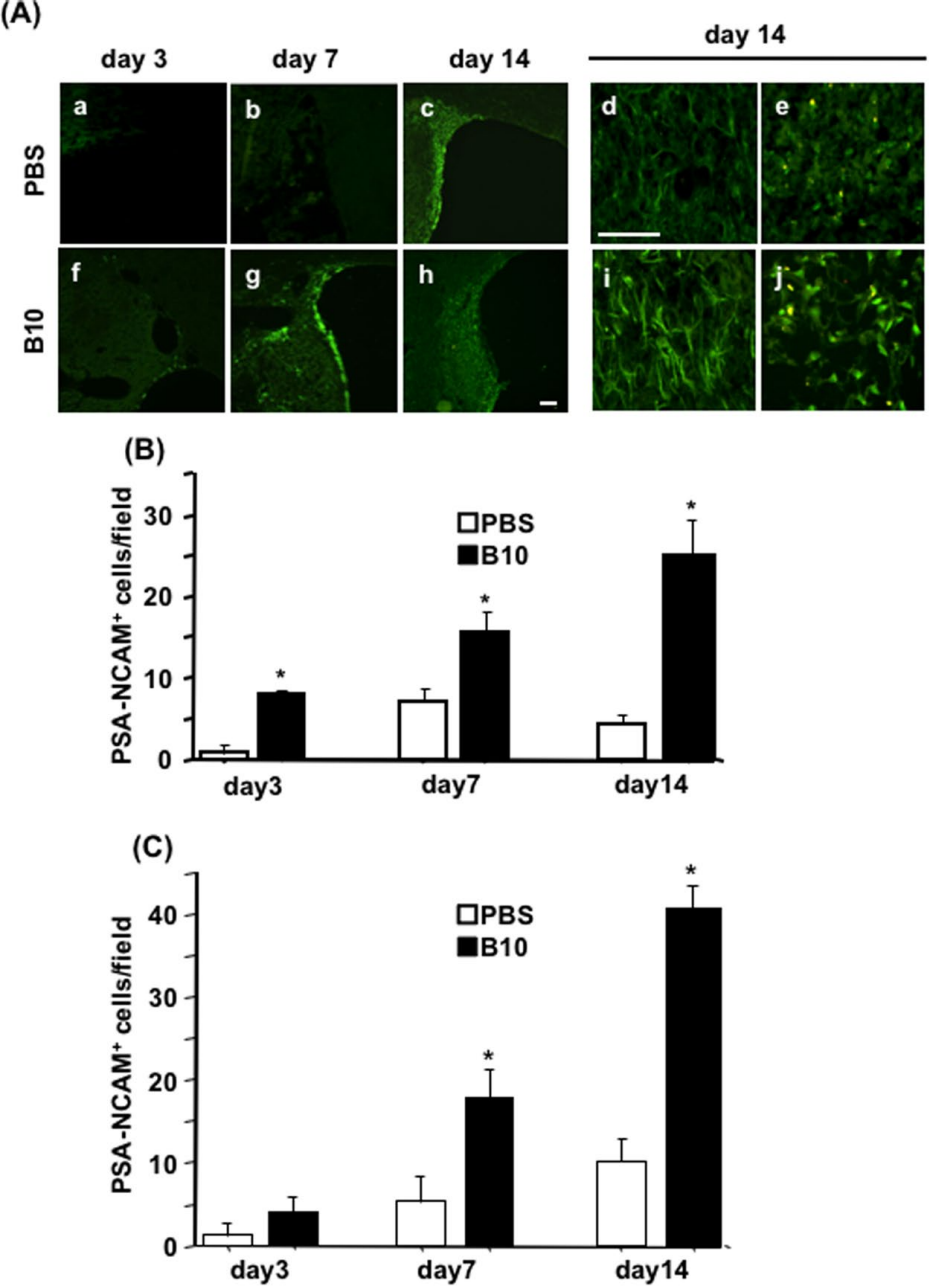
Temporal and spatial distribution of PSA-NCAM^+^ neuronal progenitors in the rat brain after MCAO. (**A**) To determine the distribution of neuronal progenitor cells in MCAO rat brains, immunofluorescence staining for PSA-NCAM was performed, as described in the Materials and Methods. The distribution of PSA-NCAM^+^ cells in the SVZ area of PBS (a–e) and B10 transplanted (f–j) rats at day 3 (a and f), day 7 (b and g) and day 14 (c and h) are shown at low magnification. The distribution and morphology of PSA-NCAM^+^ cells at day 14 in penumbra and core of PBS (d and e) and B10 transplanted (i and j) rats are shown at higher magnification. Scale bar is 100 μm in (a–c and f–h), and 20 μm in (d,e,i and j). PSA-NCAM positive cells in penumbra and core regions at day 3, 7 and 14 after MCAO were counted at X400 magnification and shown in (**B**,**C**), respectively. Numerical data are presented here as mean ± standard error of means (n = 5). Statistical significance was denoted as follows: *p < 0.01 vs PBS group of same time point.

First, the effects of B10 transplantation on the distribution of migrating neuronal progenitor cells (NPCs) were evaluated using PSA-NCAM as a marker. PSA-NCAM-immunostaining results demonstrated the presence of a few immunoreactive cells in the SVZ on the ipsilateral side of B10 transplanted MCAO rat at day 3, which was gradually increased at day 7 (Fig. 1Af–g), and the number was decreased at day 14. In the case of PBS-control MCAO rats, PSA-NCAM-positive cells were not detectable at day 3, then they appeared at day 7 and the number was increased at day 14 (Fig. 1Aa–c). Further investigations revealed that, PSA-NCAM positive cells were migrated to the penumbra and the core regions in both PBS-control and B10 transplanted MCAO rats (Fig. 1Ad,e and Ai–j). In the case of B10 transplanted MCAO rats, the cells were detectable in the lesion area from day 3, and gradually increased in number until day 14 (Fig. 1B,C). Conversely, in PBS-control rats, PSA-NCAM positive cells in the lesion area were barely detectable at day 3, and the number was increased gradually until day 14, at least in the core area (Fig. 1B,C). Importantly, the cell number in the penumbra and core areas was higher in B10 transplanted rats at all time points (Fig. 1B,C). On the contralateral side, no significant changes of PSA-NCAM positive cell number was observed between B10 transplanted and PBS-control groups (data not shown).

### Identification of PSA-NCAM positive cells in MCAO rat brain

**Figure 2:**
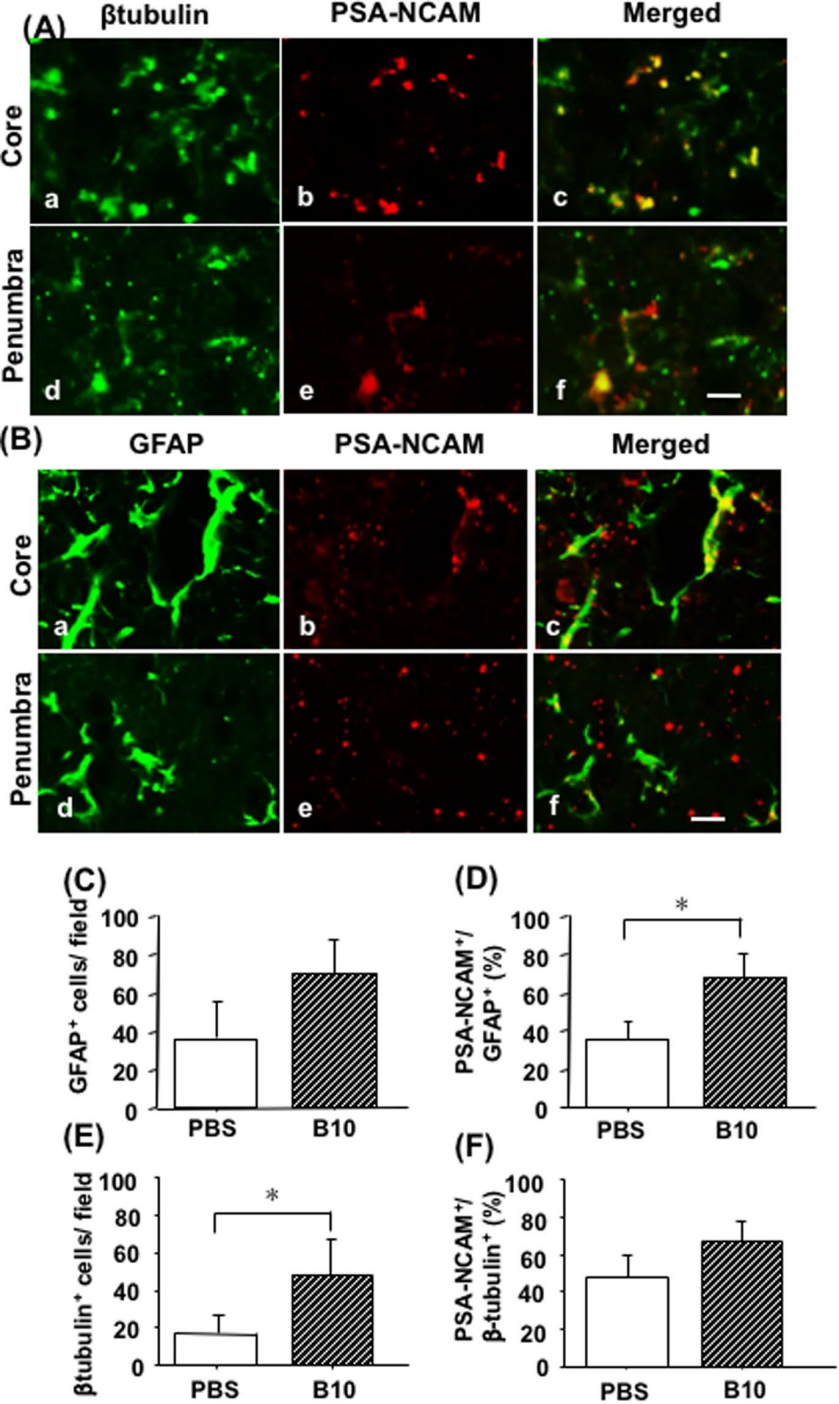
Differentiation potentials of PSA-NCAM positive neuronal progenitors in rat brains after MCAO. To determine the differentiation potentials of PSA-NCAM positive cells, double immunofluorescence staining of PSA-NCAM and cell type specific markers (GFAP for astrocytes, and β-tubulin for neuronal markers) were done, as described in the Materials and Methods. Representative photomicrographs of β-tubulin (a and d) and PSA-NCAM (b and e) double immunofluorescence staining of day 14 MCAO rat brains, and their merged pictures (c and f) are shown in (**A**), where (a–c) are photomicrographs of core, and (d–f) are of penumbra area. Representative photomicrographs of GFAP (a and d) and PSA-NCAM (b and e) double immunofluorescence staining of day 14 MCAO rat brains, and their merged pictures (c and f) are shown in (**B**), where (a–c) are photomicrographs of core, and (d–f) are of penumbra area. GFAP^+^ and β-tubulin^+^ cells at day 14 were counted at X400 magnification and shown in (**C**,**E**), respectively. Double positive cells were counted and their percentage over total GFAP and β-tubulin were calculated, and shown in (**D**,**F**), respectively. Numerical data are presented here as mean ± standard error of means (n = 5). Statistical significance was denoted as follows: *p < 0.01 vs PBS group. Scale bar = 20 μm.

In order to investigate about the differentiation potentials of NPCs in the penumbra and core region of day 14 MCAO rat models, double immunofluorescence staining for PSA-NCAM and cell type-specific markers were performed. In PBS-control MCAO rats, GFAP positive astrocytes that showed PSA-NCAM immunoreactivity was mainly found in the core area (Fig. 2Ba–c). However, β-tubulin positive neurons showed PSA-NCAM immunoreactivity in both core and penumbra regions (Fig. 2A). The distribution pattern of PSA-NCAM positive astrocytes and neurons in B10 transplanted rats were similar to PBS-control MCAO rats. Counting the cells in the lesion areas demonstrated that the number of both GFAP positive astrocytes and β-tubulin positive neurons was increased in B10 transplanted group; however, the difference of astrocyte cell number did not reach a statistically significant level (Fig. 2C,E). Interestingly, the percentage of PSA-NCAM positive astrocytes, but not β-tubulin positive neurons, was significantly increased in B10 transplanted group (Fig. 2D,F).

### Differentiation potentials of transplanted B10 cells in MCAO rat brains

**Figure 3:**
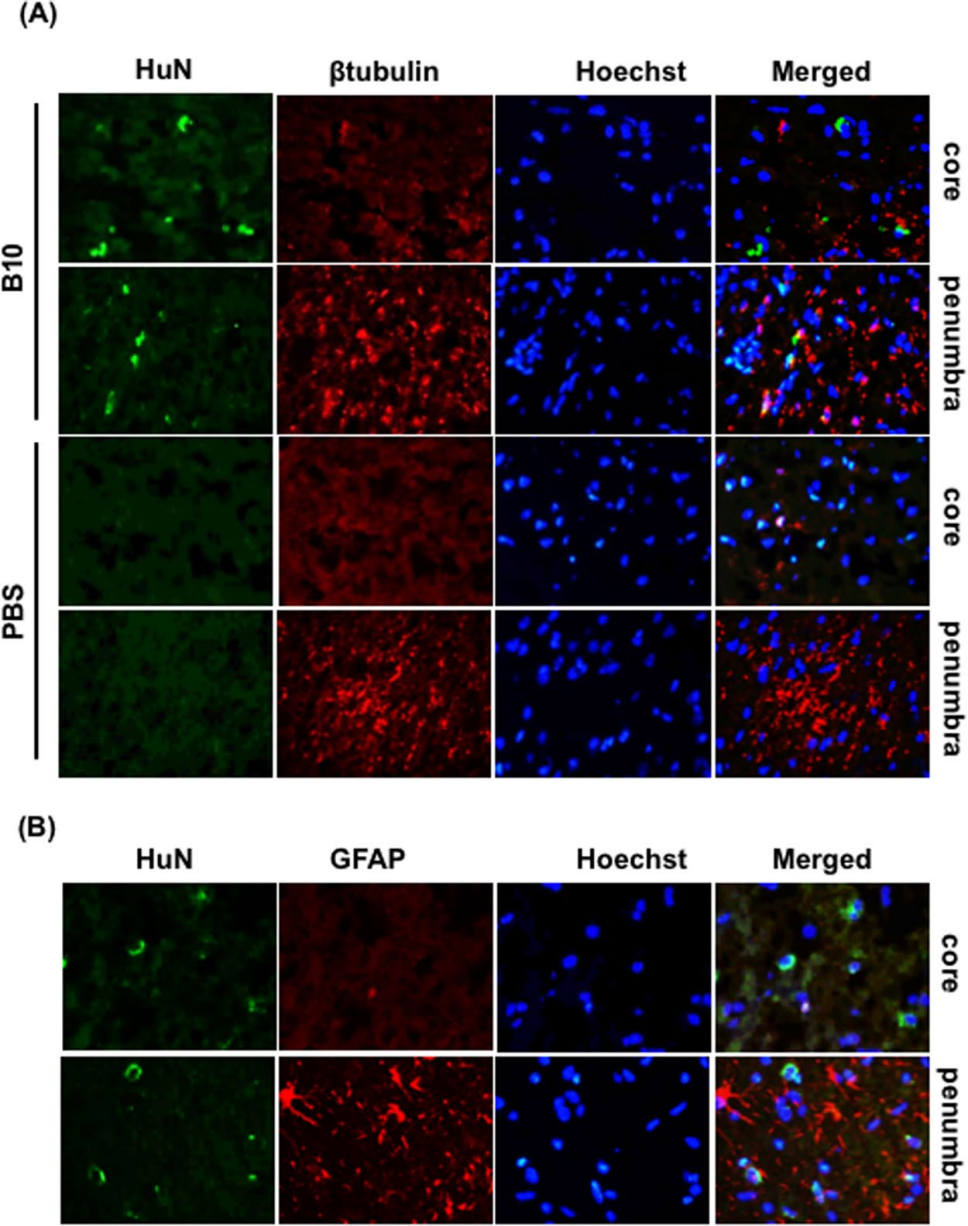
Differentiation potentials of transplanted B10 cells in MCAO rat brain. To determine the differentiation potentials of B10 cells in MCAO rat brain, double immunofluorescence staining of human nuclei (HuN) and cell type specific markers (GFAP for astrocytes, and β-tubulin for neuronal markers) were done in day 3 MCAO rats. Representative photomicrographs of HuN and β-tubulin double immunofluorescence staining of PBS treated and B10 transplanted rats are shown in (**A**), and double immunofluorescence staining of HuN and GFAP of B10 transplanted rats are shown in (**B**). Hoechst staining was done to identify the nuclei.

Next, we investigated whether transplanted cells could have the ability to differentiate to neurons or astrocytes. In our previous study, we found that transplanted B10 cells were easily detectable at day 3 (48 h after transplantation), and persisted in the lesion areas up to day 5 after MCAO^19,32^. Hence, we examined the differentiation in day 3 MCAO model using cell type specific markers. At day 3, very few GFAP and β-tubulin positive cells were found in the core region of both PBS- and B10 transplanted rat brains. Double immunofluorescence staining results showed that human nuclei (human origin B10 marker) did not co-localize with β-tubulin (neuron marker) or GFAP (astrocyte marker) 3 days after MCAO (Fig. 3A,B).

### Effects of B10 transplantation on mRNA expression of endogenous migration regulators in MCAO rat brain

**Figure 4:**
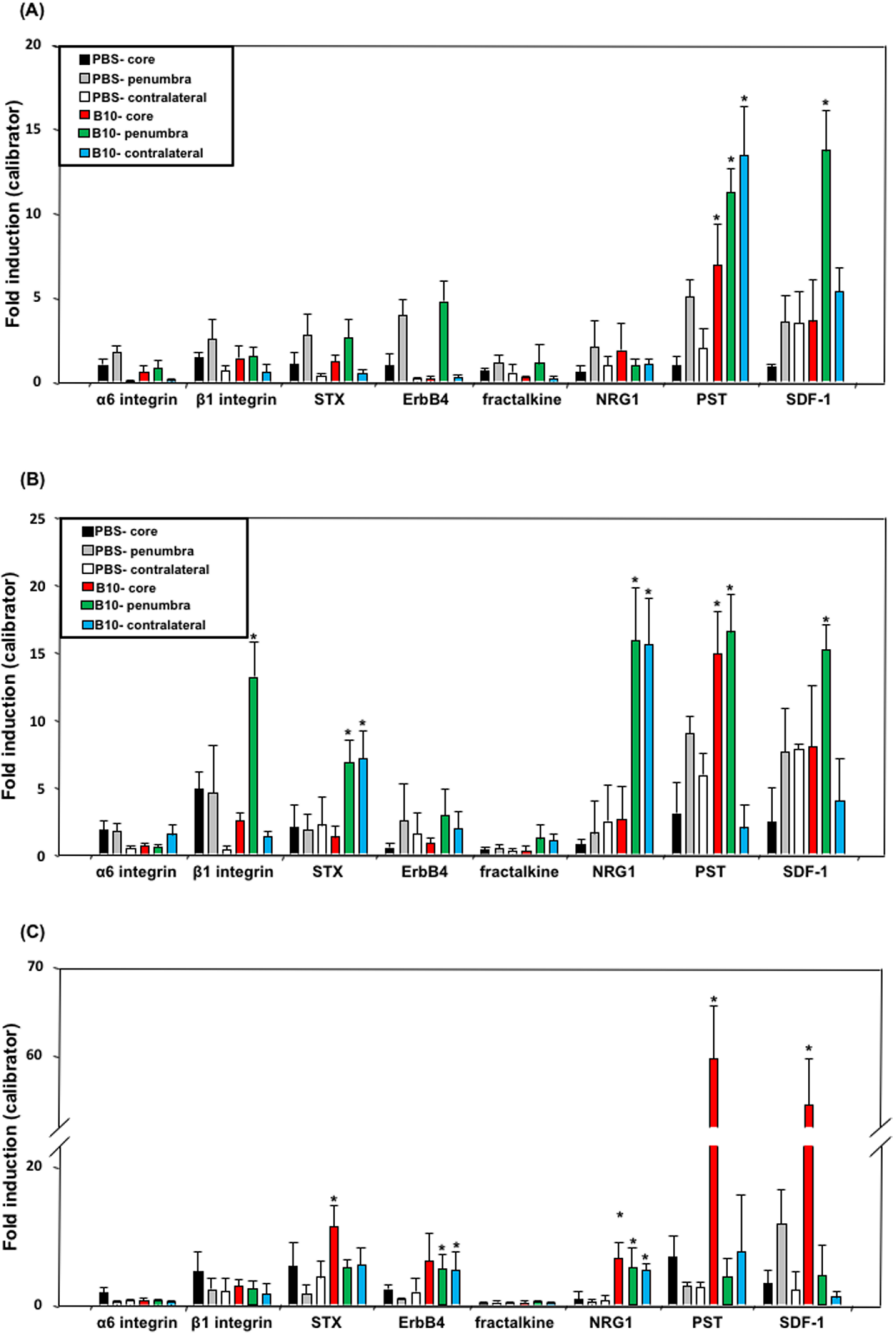
Effect of B10 transplantation on mRNA expression of endogenous migration regulators in MCAO rat brain. To determine the effects of B10 transplantation on the regulation of neuronal progenitor’s migration, the mRNA expression of migration regulators was analyzed. Total RNA was isolated from core, penumbra and contralateral cortex of PBS-treated and B10-transplanted rat at day 3 (**A**), day 7 (**B**) and day 14 (**C**) after MCAO, and mRNA expression of migration regulators was analyzed by a real-time PCR system, as described in Materials and Methods. The PCR data is presented as mRNA level relative to a calibrator sample, and expressed as mean ± SD of 5 samples in a group. GAPDH mRNA was used as loading control. Statistical significance was denoted as follows: *p < 0.01 vs PBS group of same area and same time point.

Next, the mRNA expression of the factors related to cell migration including α6-integrin, β1-integrin, STX, ErbB4, fractalkine, NRG1, PST and SDF-1 was analyzed in the brain tissue of B10 transplanted and PBS-control rats at 3, 7 and 14 days after MCAO, by real time RT-PCR. The results showed that compared to PBS-control, PST mRNA level was increased in the core, penumbra and contralateral cortex at day 3 of B10-transplanted rats (Fig. 4A). The mRNA level in the contralateral cortex returned to similar level as PBS-control at day 7, and in the penumbra at day 14 (Fig. 3B,C). SDF-1 mRNA expression was found to be increased in the penumbra at day 3 and 7, and in the core at day 14 in B10 transplanted group (Fig. 3A–C). In the case of STX, the mRNA level was found to be increased in the penumbra and contralateral cortex at day 7 and in the core at day 14 (Fig. 3A–C). The effect of B10 transplantation on NRG1 mRNA was started to observe from day 7, the level was increased in the penumbra and contralateral cortex at day 7, and in the core at day 14. However, the mRNA of ErbB4, a receptor for NGR1, was increased only at day 14 in the penumbra and contralateral cortex.

### Quantification and identification of SDF-1 expressing cells in MCAO rats

**Figure 5:**
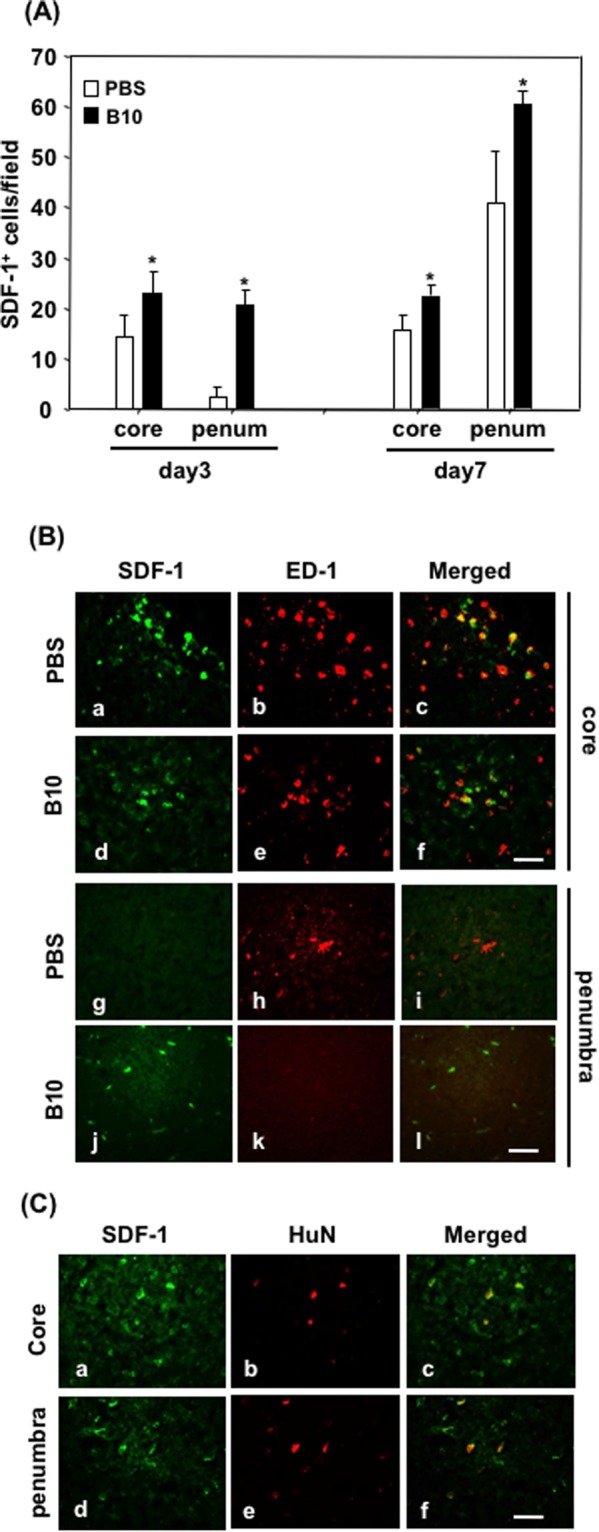
Quantification and identification of SDF-1 expressing cell types in rat brains after MCAO. (**A**) After immunofluorescence staining of SDF-1, positive cells were counted in 5 random field of designated areas at X400 magnification. Three tissue sections of 2 mm apart were used to count the cells, and the average was considered as positive cell number of that rat. The data presented here as average ± SD (n = 5), and the statistical significance was evaluated by one-way ANOVA followed by Student-Newman-Keuls Post hoc analysis. *p < 0.05. (**B**) To determine the localization, double immunofluorescence staining of SDF-1 and macrophage/microglia marker ED1 was done, as described in the Materials and Methods. Representative photomicrographs of SDF-1 (a,d,g and j) and ED-1 (b,e,h and k), and their merged picture (c,f,i and l) in the core (a–f) and penumbra (g–l) areas of PBS-control (a–c and g–i) and B10-transplanted (d–f and j–l) rats are shown. (**B**) To determine SDF-1 expression in transplanted B10 cells, double immunofluorescence staining of SDF-1 and human nuclei (to identify human origin B10 cells) was done in B10 transplanted rats 3 days after MCAO. Representative photomicrographs of SDF-1 (a and d), human nuclei (b and e) and their merged pictures (c and f) in the core (a–c) and penumbra (d–f) are shown. Scale bar = 50 μM.

Since the mRNA level of SDF-1 mRNA was consistently increased in B10 transplanted group throughout the observation period, we sought to quantify and identify the cells that expressed it in MCAO condition. Immunostaining results showed that SDF-1 positive cell number was significantly increased in B10 transplanted group both at day 3 and day 7 in the core and penumbra region (Fig. 5A). Double immunofluorescence results demonstrated that SDF-1 was expressed in ED-1positive macrophage/microglia in the core region at day 3 in both PBS-control and B10-transplanted MCAO groups (Fig. 5Aa–f). In the penumbra of PBS-control MCAO rats, SDF-1 expressing cells were barely detected at day 3. In the case of B10-transplanted group, ED-1 positive macrophage/microglia was not detectable in penumbra at this time point, whereas SDF-1 positive cells were found. Double immunofluorescence result showed that transplanted B10 cells expressed SDF-1 in both core and penumbra area (Fig. 5B).

### B10-derived SDF-1 increased migration of a neuronal cell line

**Figure 6:**
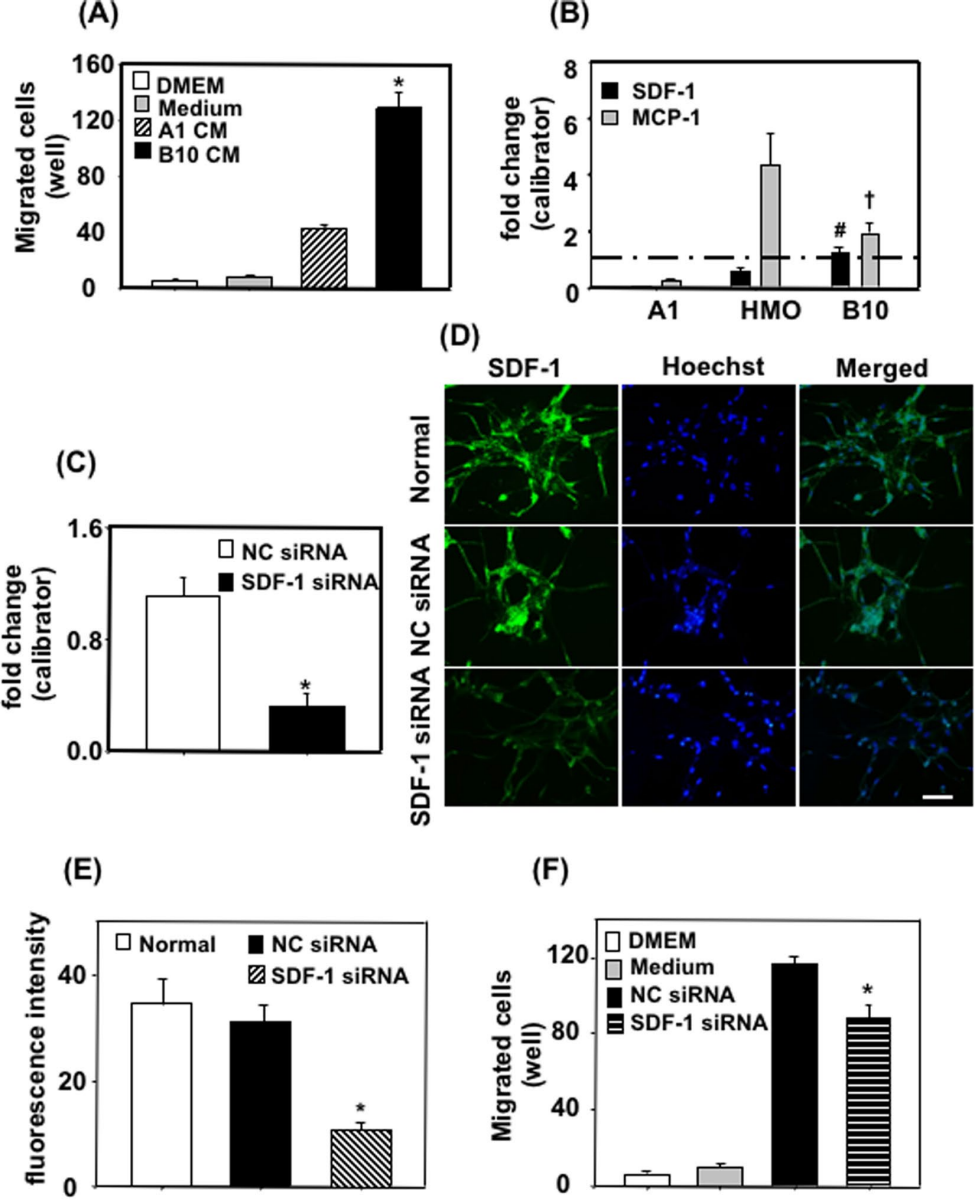
Effects of B10 cells on neuronal cell migration in culture. (**A**) To assess the effects of B10-secreted molecules on neuronal migration, B10 cells were cultured to confluency in complete medium. Then the medium was changed to 0.5% FBS containing DMEM, cultured for further 24 h and the culture supernatant was used for A1 migration assay. DMEM-only, DMEM containing 0.5% FBS (medium) or A1 culture supernatant was used as control. *p < 0.05 vs control. (**B**) The mRNA levels of chemokines for neural progenitor cells in B10, A1 and HMO6 were analyzed by real time PCR. The cells were incubated in 0.5% FBS containing DMEM for 24 h, and total RNA was isolated. RNA of B10 native culture was used as a calibrator. ^#^*p* < 0.005 vs SDF-1 mRNA in A1 or HMO; ^†^*p* < 0.05 vs MCP-1 mRNA in A1 or HMO. (**C**) To inhibit SDF-1 mRNA expression, B10 cells were transfected with gene specific siRNA, negative control siRNA (NC siRNA), and cultured for 48 h. Then SDF-1 mRNA levels were measured by real time PCR, where RNA of a B10 native culture was used as calibrator. *p < 0.05 vs NC siRNA. (**D**,**E**) To further evaluate the siRNA mediate inhibition, B10 cells were stained with anti-SDF-1 antibody. Representative photomicrographs of SDF-1 immunocytochemistry are shown in (**D**), and fluorescence quantification data in (**E**). *p < 0.05 vs normal culture or NC siRNA condition. After 48 h, medium was changed to 0.5% FBS containing DMEM, and cultured for further 24 h. Then the culture supernatant was collected and used for migration assay (**F**). *p < 0.05 vs NC siRNA culture supernatant.

To investigate about the role of B10 on neural progenitor cell migration, we checked the effects of B10 culture supernatant on the migration of a neuronal line (A1) using a Boyden chamber migration assay system. Migration assay results showed that B10 culture supernatant significantly increased A1 cell migration compared to control medium or A1 cell culture supernatant (Fig. 6A).

Previous reports have shown that SDF-1 and MCP-1 play an important role in the migration of neuronal progenitor cells^33,34^. Since B10 culture supernatant increased A1 neuronal cell migration, we checked the mRNA expression of SDF-1 and MCP-1 in B10 cells. Real time PCR results showed that SDF-1 mRNA expression was significantly higher in B10 cells than a human microglia cell line (HMO6) or A1 cells (Fig. 6B). Moreover, MCP-1 mRNA expression in B10 cells was higher than A1 cells (Fig. 6B). To investigate further about the role of SDF-1 on B10 culture supernatant-mediated A1 migration, SDF-1 gene silencing was done in B10 by transfecting siRNA (Fig. 6C–E). Then the culture supernatant was used for A1 migration assay. The results demonstrated that inhibiting production in B10 cells by SDF-1 specific siRNA significantly inhibited B10 culture supernatant-mediated A1 migration compared to negative control siRNA transfected B10 culture supernatant (Fig. 6F).

## Discussion

Experimental evidence has established that ischemic insults can induce neurogenesis in damaged brains^35^, indicating the fact that adult brain retains the ability to repair itself. However, this ability might not be sufficient enough to cause a significant recovery, and boosting up the effect could be a good strategy for stroke therapy^36^. In this study, we have demonstrated that a mesenchymal stem cell line (B10) transplantation not only increased the proliferation and migration of neuronal progenitors, but also started it at an earlier time. In a previous study it has been shown that MSC transplantation can enhance oligodendrocyte progenitor proliferation in cerebral ischemia model^37^, suggesting its influence on endogenous stem cells. In our previous studies, we have demonstrated that transplanted B10 cells have the ability to migrate to ischemic area of rat brains, and detectable up to 5 days after MCAO. Although B10 cells transiently present only at an earlier time of pathology, we found their effects continued for a prolonged period of time (up 14 days). B10 transplantation can increase several growth factor expression including bFGF, IGF and EGF^19^, which have neurotrophic and neuroprotective properties. Growth factors play an important role in neuroprotection, as demonstrated by a recent report that MSC co-expressing BDNF and VEGF shows improved neuroprotection efficacy^38^. Moreover, B10 have the capability to modulate neuroinflammation in a way that have a protective effect on neurons^19^. Numerous studies including ours have shown the beneficial effects of the regulation of neuroinflammation and growth factors expression in cerebral ischemia^18,19,32,39^. Combining the findings of previous reports with the current study, a coordinated action of the regulation of neuroinflammation, neuroprotection by growth factors and neurogenesis and migration might cause the functional improvement in B10 transplanted stroke animals. Hence, MSC transplantation could be a good strategy for overall restorative therapy in cerebral ischemic condition.

Previous studies have showed that PSA-NCAM is expressed in neuronal progenitor cells, and their expression is restricted to the regions where the adult brain retain new neuron generation property. Doublecortin (DCX) or Ki67 can also be used as markers for proliferating neuronal progenitor cells. However, Ki67 is a general marker for proliferating cells, and DCX is shown to be expressed mature astrocytes also. Hence, PSA-NCAM could be a better marker for neuronal progenitor cells. Polysialylation of neural cell adhesion molecule (NCAM) is suggested to enhance the migration of neural progenitors towards migration guidance cues including basic FGF and BDNF^40^. In this study, PSA-NCAM positive neural stem cells appeared as early as 3 days after MCAO in B10 transplanted group, suggesting that polysialylation might be an important factor of the migration of neural progenitors. Our real time PCR data showed that B10 transplantation caused a sustained increase of PST expression from day 3, whereas another polysialylation enzyme STX expression was increased from day 7. Hence, B10-induced PST-mediated polysialylation possibly have a bigger role in the migration of neural progenitors, at least during earlier time period. Moreover, our previous study showed that B10 transplantation increases the expression of growth factors such as BDNF and basic FGF after 7 days of MCAO, which are shown to be involved in PSA-NCAM dependent migration of neuronal progenitors^19,40^. Therefore, combined effects of increased growth factors expression and polysialylation of NCAM might cause an increase of neuronal progenitor migration towards the lesion areas, at least during later phase of disease pathology.

In a previous study, SDF-1 and its receptor, CXCR4 have been demonstrated to direct the migration of stem cells in ischemic lesions^41^. In this study, B10 transplantation increased SDF-1 mRNA level from very early time point that persisted until 14 days after MCAO. Immunostaining data demonstrated that SDF-1 is mainly produced in macrophage/microglia in MCAO condition. Interestingly, many transplanted B10 also expressed SDF-1, suggesting the important role of transplanted cells B10 cells on neuronal progenitor migration. Among the chemokines, the expression of SDF-1 was found to be high in B10 cell culture, even higher than in a microglia cell line (HMO6). *In vitro* studies also confirmed the effects of B10 on neuronal cell migration. Gene silencing studies showed that SDF-1 inhibition significantly, but partially inhibited neuronal cell migration. The reason of partial inhibition might be that B10 could produce some other chemokine for A1 cell migration. Moreover, A1 cell is not a neural progenitor but an immature-type neuron. Hence, the migration regulation of neural progenitors and immature neurons are different. The transplanted B10 cells were found to exist in MCAO rat brains for maximum 5 days, indicating the importance of B10 cell derived SDF-1 for early migration of neuronal progenitors.

Neuronal progenitor is demonstrated to have the ability to differentiate to both neurons and glial cells^42,43^. Our immunostaining results have shown that both GFAP positive astrocytes and β-tubulin positive neurons are positive for PSA-NCAM. These results are suggesting that neuronal progenitors that migrated from SVZ area have the potential to differentiate to both astrocytes and neurons^44^. Importantly, β-tubulin positive neuronal number was significantly increased in B10 transplanted group, although the percentage of PSA-NCAM positive neuron number was not significantly different from PBS treated groups at day 14. As PSA-NCAM level tends to decrease during the maturation of neurons^45^, our results suggested that migrated neuronal progenitors are already differentiated to advanced stage leading decreased level of polysialylation of NCAM. On the contrary, PCA-NCAM positive astrocytes were significantly increased in B10 transplanted rat brains without increasing total astrocyte number. This result is suggesting that many astrocytes are derived from neuronal progenitors in this condition, although their differentiation process might not complete at this stage like neurons. As the total astrocytes number is not different, the neurotrophic and neuroprotective function of astrocytes might be similar in B10 transplanted and PBS treated brains. However, a functional difference between resident astrocytes and newly differentiated astrocytes could exist, which is interesting to investigate in stroke condition.

In conclusion, this study suggests that B10 transplantation increased the proliferation and migration of neuronal progenitors by polysialylation of NCAM, and regulated the expression of ErbB4 and SDF-1. Such regulation of regenerative system might be important for the functional neurological improvement that we observed in MSC-based management of stroke.

## Electronic supplementary material

Supplementary Dataset 1

## Data Availability

All additional data of this study and experimental procedures is abailable upon request to corresponding author.
