# Supplementary material for: Transplantation of a bone marrow mesenchymal stem cell line increases neuronal progenitor cell migration in a cerebral ischemia animal model
Source: Sci Rep. 2018 Oct 8;8:14951. doi: 10.1038/s41598-018-33030-9 (PMC6175868; doi:10.1038/s41598-018-33030-9)
Supplement: Supplementary file 1 — Supplementary Dataset 1 [file 41598_2018_33030_MOESM1_ESM.pdf]

Transplantation of a bone barrow mesenchymal stem cell line increases neuronal progenitor cell migration in a cerebral ischemia animal model

Yuri Shiota[1], Atsushi Nagai[1, 2*], Seiji Mishima[1],Abdullah Md. Sheikh[2], Shingo Mitaki[3], Shozo Yano[2], Haque Md. Ahsanul[2], Shotai Kobayashi[4], Shuhei Yamaguchi[3]

[1]Clinical Laboratory Division, Shimane University Hospital, 89-1 Enya Cho, Izumo 693-8501, Japan

[2]Department of Laboratory Medicine, Shimane University School of Medicine, 89-1 Enya Cho, Izumo 693-8501, Japan

[3]Department of Internal Medicine III, Shimane University School of Medicine, 89-1 Enya Cho, Izumo 693-8501, Japan

[4]Shimane University, 89-1 Enya Cho, Izumo 693-8501, Japan

Address correspondence and reprint requests to:

Dr. Atsushi Nagai,

Department of Laboratory Medicine, Shimane University Faculty of Medicine, 89-1 Enya-cho, Izumo 693-8501, Japan.

Tel: 81-853-20-2409; Fax: 81-853-20-2409;

E-mail: anagai@med.shimane-u.ac.jp

Supplemental Table 1

Modified neurological scoring system (mNSS)

Motor test			
	Raising by the tail: maximum points = 3<br>Inability to-		
		flexion of forelimb: point 1	
		flexion of hind limb: point 1	
		head moved >10 in vertical axis within 30 s: point 1	
	Placing rat on the floor: maximum points = 3		
		Normal walk: point 0	
		Inability to walk strait: point 1	
		Circling towards paresis side: point 2	
		Fall down to the paretic side: point 3	
Sensory test: maximum points = 6			
	Visual test: point 1point		
	Tactile test: point 2 points		
	Proprioceptive test (deep sensation): 3 points		
Balance beam test: maximum points = 6			
	Balance with steady posture: 0 point		
	Grasp the side of the beam: 1 point		
	Hug the beam, and 1 limb fall down from the beam: 2 points		
	Hug the beam, and 2 limbs fall down from the beam, or spin on beam > 60 sec: 3 points		
	Attempt to balance but fall down > 40 sec: 4 points		
	Attempt to balance but fall down > 20 sec: 5 points		
	Fall down, no attempt to balance (<20 sec): 6 points		
Absence of reflexes, and abnormal movements: maximum points = 4			
	Corneal reflex (eye blinks when lightly touching the cornea with cotton): 1 point		
	Startle reflex (motor response to a brief noise from snapping a		

	clipboard paper): 1 point
	Pinna reflex (head shakes when touching the auditory meatus): 1 point
	Seizures, myoclonus, myodystonia: 1 point
	Maximum total points = 22

Supplemental Table 2

Primer sets used for real time PCR

Gene name	Forward primer	Reverse primer	Gene ID
α6 integrin	caaggcagatggagtaatgtgaag	cgattccaaacatcgagtctttg	NM_053725
β1 integrin	gcatctgaaccatgactgattctc	cagcaggactgttctttgattctg	NM_017022
STX	ggagtgggtcaatgctctcatc	cttttgatgtggactttgtt	NM_057156
ErbB4	cgggccattccactttacc	ccatgatgaccaggatgaagag	AF041838
Fractalkine	acctcggcatgacgaaatg	cctggttcagttgatagtggatgag	AF030358
NRG1	ttacacttccacagcccatcac	ctctccgtgtgcccattactc	NM_031588
PST	cggaggcaaggctcagaac	ttgatgagtgggatacccttcac	X52833
SDF-1	ccagagccaacgtcaaacatc	acacttgtctgttgttgcttttcag	AF209976
GAPDH	cagcctcgtcatagacaagatg	aaggcagccctggtaacca	NM_017008
